# TNF*α* deficiency results in increased IL-1*β* in an early onset of spontaneous murine colitis

**DOI:** 10.1038/cddis.2017.397

**Published:** 2017-08-10

**Authors:** S De Santis, D Kunde, V Galleggiante, M Liso, L Scandiffio, G Serino, A Pinto, P Campiglia, R Sorrentino, E Cavalcanti, A Santino, M L Caruso, R Eri, M Chieppa

**Affiliations:** 1 National Institute of Gastroenterology 'S. de Bellis', Research Hospital, Castellana Grotte, Bari 70013, Italy; 2 Institute of Sciences of Food Production C.N.R., Unit of Lecce, via Monteroni, Lecce 73100, Italy; 3 Mucosal Biology, School of Health Sciences, University of Tasmania, Launceston, TAS, Australia; 4 Department of Pharmacy, Faculty of Pharmacy and Medicine, University of Salerno, Fisciano (SA), Italy

## Abstract

Inflammatory bowel disease (Crohn’s disease (CD) and ulcerative colitis (UC)) is a multifactorial disease resulting from immune dysregulation in the gut. The underlying colitis is characterized by high levels of inflammatory cytokines, including TNF*α*. Biological intervention for IBD patients using anti-TNF*α* antibodies is often an effective therapeutic solution. However, TNF*α* neutralization fails to induce remission in a subgroup of IBD patients, primarily in UC patients. There is a dearth of suitable animal models representing TNF*α* non-responders. Here we have combined one of the best UC models currently available, namely *Winnie* and the TNF*α*KO mouse to generate a TNF*α*-deficient *Winnie* to study early onset colitis. The induced TNF*α* deficiency with underlying colitis does not influence general health (viability and body weight) or clinical parameters (colon weight, colon length and histological colitis) when compared with the *Winnie* genotype alone. The molecular characterization resulted in identification of *Il1β* as the major elevated cytokine during early phases of colitis. Further, *in vitro* functional assay using bone marrow-derived dendritic cells confirmed IL-1*β* as the major cytokine released in the absence of TNF*α*. This study has generated a successful model of colitis that remains TNF*α* non-responsive and has demonstrated that IL-1*β* expression is a major pathway for the progression of colitis in this system. These data also suggest that IL-1*β* can be a potential target for clinical intervention of UC patients who fail to respond to TNF*α* neutralization.

Inflammatory bowel diseases (IBD) include Crohn’s disease (CD) and ulcerative colitis (UC), which are both chronic relapsing inflammatory disorders of the gastro-intestinal tract.^1^ The mucosal immune system is dynamically regulated to a state of tolerance to luminal antigens including commensal bacteria and food-derived antigens. However, breaches of mucosal immune tolerance can occur due to both environmental and genetic factors resulting in perturbed intestinal homeostasis. Several different events can trigger inflammatory responses that may result in chronic inflammation and pathological changes associated with IBD. A common ground for this multifactorial disorder is an increased production of diverse panel of cytokines,^2, 3^ some of which are pro-inflammatory and hence targets for therapeutic blockade.^4^ Among the cytokine milieu dysregulated in IBD-like chronic inflammatory conditions, tumor necrosis factor *α* (TNF*α*) is a major pro-inflammatory cytokine that drives downstream immune responses resulting in IBD. Colonic macrophages are reported to be the major source of TNF*α* in active IBD.^5^ Studies of macrophages isolated from the colon of patients with active IBD have reported elevated TNF*α* production.^6, 7^ Additionally, TNF*α* is also produced by numerous other immune cells such as T and B lymphocytes,^8, 9^ intestinal epithelial cells^10^ and several other cells.^11^ It is well known that TNF*α* polymorphisms result in increased pathogenic TNF*α*-driven intestinal gut pathology in CD.^12^ Clinical investigations have led to the discovery of anti-TNF*α* monoclonal antibodies (Infliximab and others) which have dramatically changed the medical approach to IBD.

Similar to CD, a subgroup of UC patients also exhibit increased TNF*α* levels in the colon. In fact, Braegger *et al.*^13^ reported the presence of TNF*α* in stool samples in such UC patients. Numerous studies have confirmed the crucial role of TNF*α* in colitis.^14, 15^ Results obtained by Corazza *et al.* were particularly relevant in exploring the role of TNF*α* in the onset of UC. Using TNF*α*KO murine models they were able to demonstrate the importance of TNF*α* production by non-T cells of the colonic mucosa in the pathogenesis of colitis.^16^ However, there is a subgroup of human IBD non-responders to anti-TNF*α* therapies, particularly in the UC cohort.^17^ Animal models have significantly contributed to the elucidation of the pathological mechanisms of IBD and to the validation of immunological targets for IBD treatment.^18^ However, using the currently available murine models we are unable to decipher the exact mechanism as to why some subgroups of UC patients do not respond to anti-TNF*α* therapies.

In order to address the lack of a model with relevance to the anti-TNF*α* non-responding UC patient group we planned to develop a viable model based on the best recently described murine model of UC currently available, namely *Winnie*. *Winnie* colitis is due to a missense mutation in the *Muc2* mucin gene^19^ resulting in spontaneous distal colitis developing as early as 5 weeks of age. In the *Winnie* mouse the colonic pathology is predominantly mediated by the dysregulation of numerous cytokines, including elevated TNF*α* similar to human UC where the intestinal inflammation is most severe in the distal colon and the disease severity increases with age.^20^ In order to remove the effect of TNF*α* in the *Winnie* spontaneous colitis, we generated *Winnie*/TNF*α* knockout to explore the axis between TNF*α* and intestinal inflammation that may resemble UC non-responders to anti-TNF*α* therapies,^21, 22^ or patients who become non-responder (secondary non-responder) in the absence of anti-Infliximab antibodies.^23^

Detailed histological and immunological assessment of early differences between *Winnie* and TNF*α* KO *Winnie* revealed that TNF*α* KO *Winnie* exacerbates pathogenesis associated with an increased inflammatory cytokines secretion, particularly IL-1*β*. Furthermore, elevated *Il1β* transcription was detectable in 5-week-old mice prior to the onset of morphological signs of UC, suggesting that this cytokine may represent a predictive biomarker for UC patients who are non-responder to anti-TNF*α* therapy and possibly an alternative target for combinatorial biological approaches.

## Results

### TNF*α* deficiency does not influence general health and clinical parameters

**Figure 1:**
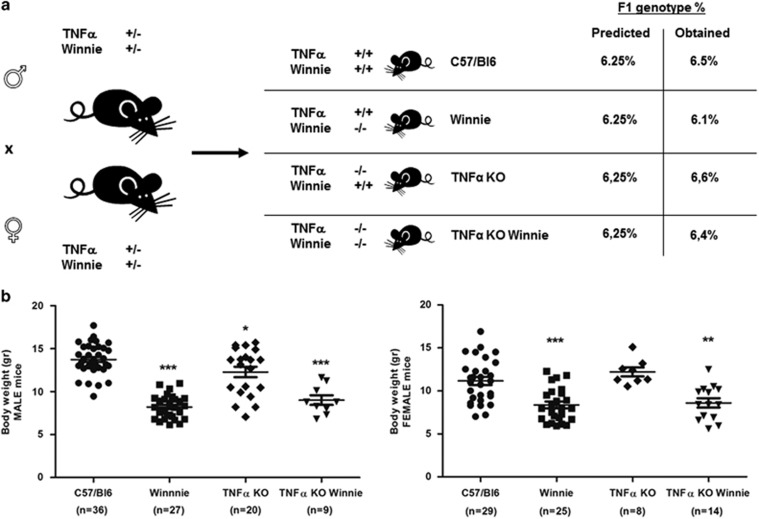
Generation and characterization of the new TNF*α* KO *Winnie* murine model. (**a**) TNF*α* KO *Winnie* mice were generated by breeding double heterozygote breeders for TNF*α* and *Winnie* genes to obtain the double mutants and all the controls from the same breeding pair. All the resulting strains were viable and no significant variation from the predicted genotype was obtained in the 4-week-old offspring. (**b**) Weight analysis of 4-week-old mice from different genotypes reveals a significant decrease in body weight in mice carrying the *Winnie* mutation independently of the TNF*α* phenotype in both males and females. Statistical evaluation between C57/BL6 mice and each murine line was performed using unpaired two-tailed Student’s *t*-test. ***P<0.001, **P<0.01, *P<0.05

TNF*α* is commonly considered the primary immunological target for IBD treatment. To investigate the axis between TNF*α* and UC onset, we used a breeding strategy based on double heterozygote breeders (Figure 1a) to obtain the double mutants and all the controls from the same breeding pair. This strategy permitted the evaluation of viability and weight of the offspring depending on the different genotypes and independently from differences related to diverse mothers. Results shown in Figure 1a demonstrate that all the resulting strains were viable and no significant variation from the predicted genotype (6.25%) was obtained in the 4-week-old offspring.

Figure 1b shows that significant differences in mouse weight could be seen in 4-week-old mice from different genotypes. Overall, *Winnie* and TNF*α* KO *Winnie* (both male and female) mice were lighter (8.28±0.23 and 8.76±0.39, respectively) than WT (C57/BL6) and TNF*α* KO controls (12.59±0.31 and 12.39±0.46, respectively). Between *Winnie* and TNF*α* KO *Winnie* there was no difference in body weight. These results show that mice carrying the *Winnie* mutation had lower body weight independently from TNF*α*.

### TNF*α* deficiency does not influence the initial development of intestinal inflammation

**Figure 2:**
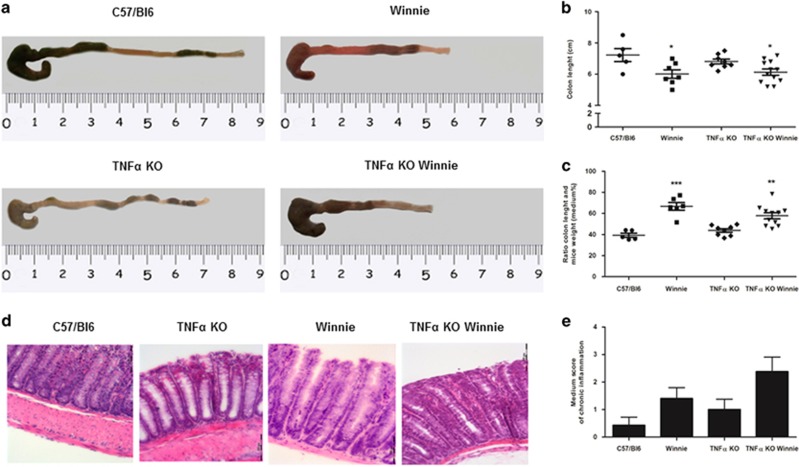
Macroscopic and histological characterization of the TNF*α* KO *Winnie*. Representative images of dissected colons (**a**) and measures of colon length (**b**) from the new transgenic line and the relative controls indicate a significant reduction in *Winnie* and TNF*α* KO *Winnie* compared with WT. This observation is strikingly evident when the colon length is adjusted for the body weight (**c**). Hematoxylin and eosin staining of 3 *μ*m sections of colon (**d**) and the average score of inflammation (**e**) show no significant differences between the TNF*α* KO *Winnie* and the controls. Statistical evaluation between C57/Bl6 mice and each murine line was performed using unpaired two-tailed Student’s *t*-test. ***P<0.001, *P<0.05

Macroscopic or histological colitis as demonstrated by colon shortening and hematoxylin and eosin (H&E) histological scoring of inflammation in C57/BL6 mice (7.22±0.42) was not significantly different from TNF*α* KO mice (6.81±0.15); however, the colon length of both groups were significantly longer than *Winnie* (6.01±0.27) and TNF*α* KO *Winnie* (6.13±0.20) if compared with WT mice (Figures 2a and b). The differences were much more striking when adjusted for body weight (Figure 2c).

Detailed histological analysis (ulceration, re-epithelization, transmural inflammation and chronic inflammation) revealed a similar picture where there was no difference in inflammatory scores between C57/BL6 mice and TNF*α* KO mice while the inflammatory scores being higher in *Winnie* and TNF*α* KO *Winnie* (Figures 2d and e). However, there was no difference between *Winnie* and TNF*α* KO *Winnie* cohorts.

### TNF*α* deficiency results in elevated expression of *Ifnγ* and *Il1β* during early onset colitis

**Figure 3:**
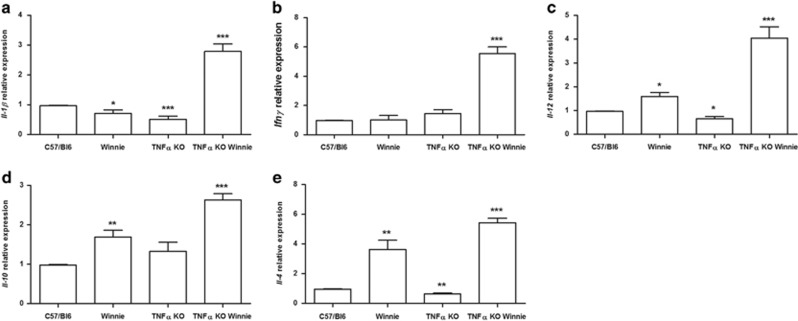
TNF*α* KO *Winnie* mice show an increased Th1 signature at the molecular level. mRNA was extracted from the colon of all the 5-week-old mice genotypes. The expression level of *Il1β, Ifnγ, Il12, Il10* and *Il4* was measured by qPCR and normalized to the housekeeping gene *Gapdh* (**a**, **b**, **c**, **d**, **e**, respectively). Bars represent mean relative expression±S.E.M. (*n*=4) for each genotype. Statistical evaluation between C57/Bl6 mice and each murine line was performed using unpaired two-tailed Student’s *t*-test. ***P<0.001, **P<0.01, *P<0.05

The cytokine expression and secretion signatures responsible for the increased inflammation in *Winnie* and TNF*α* KO *Winnie* were investigated to identify any potential molecular mechanisms mediated by TNF*α* during early onset colitis. We employed a panel of cytokine markers of Th1 (*Il1β*, *Ifnγ* and *Il12*), Th2 (*Il4*, *Il10*), Th17 (*Il17a*) and T-regs (*Foxp3*) populations to specifically quantify the relative gene expression across all cohorts by qPCR. Relative expression analysis revealed a striking elevation of *Il1β* (Figure 3a) and *Ifnγ* (Figure 3b) in TNF*α* KO *Winnie* mice compared with all other mice strains. Strikingly, in the *Winnie* background, the absence of TNF*α* leads to a molecular pathway that results in a pattern suggestive of an increased Th1 signature. The Th1-promoting cytokine *Il12* (Figure 3c) was also upregulated in TNF*α* KO *Winnie*, reinforcing the elevated *Ifnγ* observed. The expression of the anti-inflammatory cytokine *Il10* was also increased in *Winnie* and TNF*α* KO *Winnie* (Figure 3d), although the ratio between *Il12* and *Il10* is higher in TNF*α* KO *Winnie*. Furthermore, both in *Winnie* and TNF*α* KO *Winnie*, we observed a higher expression of the Th2 cytokine *Il4* (Figure 3e) compared with controls. The expression of *Il17a* and *Foxp3* was also evaluated, but results were below detection.

**Figure 4:**
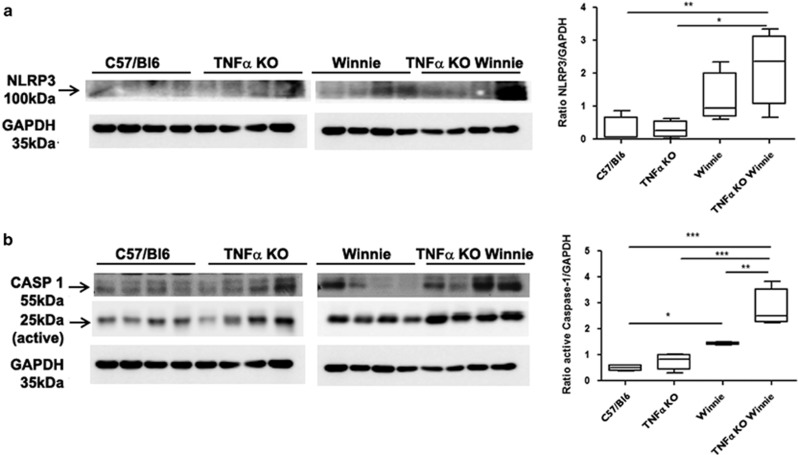
Ileum homogenates show higher expression of NLRP3 and active form of caspase-1 in TNF*α* KO *Winnie*. Western blot analyses showed that the expression of NLRP3 (**a**) was higher in both Winnie and TNF*α* KO *Winnie* mice. Similarly, (**b**) the active form of caspase-1 (25 kDa) was significantly higher in both Winnie and TNF*α* KO *Winnie* mice. Quantitative data (bar graphs) were obtained by using ImageJ (NIH, USA) software. Statistical analysis was performed by using one-way ANOVA followed by Tukey’s multiple comparison as post-test. ***P<0.001, **P<0.01, *P<0.05

**Figure 5:**
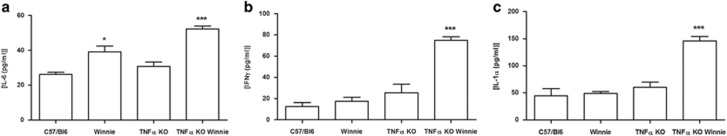
TNF*α* KO *Winnie* mice secrete high levels of circulating inflammatory cytokines. Circulating levels of IL-6, IFN*γ* and IL-1α (**a**, **b** and **c**, respectively) were determined by Bead-based Multiplex assay from serum samples of 5-week-old TNF*α* KO *Winnie* and all the control lines. Bars represent mean cytokines concentration±S.E.M. (*n*=4). Statistical evaluation between C57/Bl6 mice and each murine line was performed using unpaired two-tailed Student’s *t*-test. ***P<0.001, *P<0.05

Additionally, we evaluated the mechanism behind TNF*α* negative feedback on IL-1*β* secretion. In particular, we compared NLRP3 and caspase-1 activation in *Winnie* and TNF*α* KO *Winnie* intestinal samples. In line with the increased IL-1*β* secretion, we observed a marked increase in the levels of NLRP3 activation in *Winnie* mice particularly in the absence of TNF*α* (Figure 4a). The level of caspase-1 activation was increased in *Winnie* compared with WT, but the absence of TNF*α* strongly affected caspase-1 activation, which resulted in approximately doubled expression levels in TNF*α* KO *Winnie* (Figure 4b). Finally, we evaluated the serum levels of cytokines of 5-week-old mice (4 mice per group) by Bead-based Multiplex assay. Among the pro/anti-inflammatory molecules analyzed, IL-6 was elevated in the serum of *Winnie* and TNF*α* KO *Winnie* compared with the control, approximately two and three times higher, respectively, (Figure 5a). IFN*γ* and IL-1*α* were elevated in the TNF*α* KO *Winnie* serum (Figures 5b and c, respectively). Serum IL-1*β* was not increased in TNF*α* KO *Winnie* (data not shown), suggesting that systemic secretion of IL-1*β* was low, perhaps due to the early age and level of colitis.

### TNF*α* modulates IL-1*β* secretion in TNF*α* KO bone marrow-derived dendritic cells

**Figure 6:**
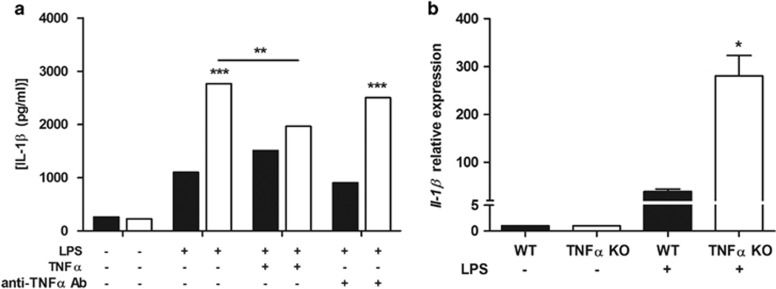
IL-1*β* upregulation in TNF*α* KO BMDCs. BMDCs were cultured from WT (black bars) and TNF*α* KO (white bars) mice, treated with TNF*α* and anti-TNF*α* antibody at day 7 and concomitantly exposed to 1 *μ*g/ml of LPS. Twenty-four hours later, the secretion of IL-1*β* was determined by ELISA (**a**). Bars represent mean cytokine concentration±S.E.M. (*n*=3). For each treatment statistical evaluation between WT and TNF*α* KO DCs was performed using two-way ANOVA and Bonferroni as post-test. For TNF*α* KO DCs treated with LPS, paired two-tailed Student’s *t-*test was conducted to compare BMDCs treated or untreated with TNF*α*. The expression level of *Il1β* was measured in WT and TNF*α* KO DCs by qPCR and normalized to the housekeeping gene *Gapdh*. Bars represent mean relative expression±S.E.M. (*n*=4) (**b**). Statistical evaluation between WT and TNF*α* KO DCs was performed using unpaired two-tailed Student’s *t*-test. *P<0.05, **P<0.01, ***P<0.001

To investigate the functional interaction between TNF*α* and IL-1*β*, we used bone marrow-derived dendritic cells (BMDCs) from wild type and TNF*α* KO mice. Figure 6a shows that 24 h after LPS administration, significantly higher IL-1*β* concentrations were detected in the supernatant of TNF*α* KO BMDCs as well as higher expression of Il-1*β* was detected in TNF*α* KO BMDCs exposed to LPS (Figure 6b). Addition of TNF*α* to TNF*α* KO BMDCs modulated IL-1*β* secretion to the same level as wild-type BMDCs (Figure 6a). We further evaluated the effect of TNF*α* by the administration of anti-TNF*α* in the BMDCs supernatant. Figure 6a shows that in wild-type BMDCs, TNF*α* depletion did not affect IL-1*β* secretion whereas TNF*α* KO BMDCs restored IL-1*β* secretion (Figure 6a) consistent with our observation in colitis.

## Discussion

In 1998 the introduction of biological therapies with blocking antibodies against TNF*α* for CD patients represented a revolutionary change for the therapy of these patients. It is now well established that the majority of IBD patients are responsive to the anti-TNF*α* therapy, but a significant percentage (up to 40% in some groups of UC) can be defined as primary non-responsive.^21, 22^ In this study we have described a potentially important mechanism linking TNF*α-*mediated inflammatory cytokine control through IL-1*β*. The murine model developed in this study incorporates a TNF*α* deficiency on a background of increased colitis that is closely representative of UC non-responders.

Murine models of IBD have been successfully used for the understanding of the biological basis of the disease and for the development of new drugs.^24^ Studies pioneered by Corazza *et al.*^16^ dissected the different role of the lymphocyte-derived TNF*α* for colitis induction. Although TNF*α* is a pivotal factor for the initiation of the inflammatory response, the absence of TNF*α* is deleterious in murine models of inflammatory disorders including experimental autoimmune encephalomyelitis^25^ and dextran sodium sulfate-induced colitis.^26^ The shortcomings of the aforementioned murine models needed to be addressed in order to investigate the role of TNF*α* in UC non-responders. Among the available models, we used the spontaneous colitis model (*Winnie*) characterized by chronic intestinal inflammation resulting from a missense mutation in the *Muc2* mucin gene.^19^ In *Winnie*, the defective mucus layer and increased intestinal permeability result in a spontaneous intestinal inflammation of the distal colon. To investigate predictive markers of TNF*α*-independent UC in a spontaneous and progressive colitis model, we crossed *Winnie* mice with TNF*α* KO (commercial line 5540) to create double KO mice (TNF*α* KO *Winnie*).

We crossed heterozygote mice to obtain all the required strains from the same breeding stock. From the genotype analysis we confirmed that all strains were viable. In our system the well-documented association between TNF*α* deficiency and low body weight was observed only for male mice even if at a lesser extent. Instead, the *Winnie* genotype, independently from TNF*α*, shows a marked reduction in the body weight. Macroscopic and histological assessment of the colon also suggest that the intestinal inflammation in the *Winnie* model was not affected by TNF*α* deficiency. Our study shows that in the absence of TNF*α* there is a significant compensatory increase in IL-1*β* production. This observation is even more interesting in the context of young mice (5-week-old) that do not yet show morphological signs of inflammation, including accumulation of inflammatory cells. Additionally, we also demonstrated that following LPS exposure, TNF*α* KO DCs produce three times the amount of IL-1*β* compared with WT DCs and that exogenous TNF*α* administration can delete the observed difference. Both these evidences point to a functional role for IL-1*β* in the pathogenesis of IBD. Recent research suggest that IL-1*β* secretion is mainly mediated by the inflammasome activation in general and NLRP3 in particular.^27^ Our data indicate that inflammasome activation may be a key aspect of protection in the absence of TNF*α*. We also observed an increased expression of *Ifnγ* and *Il4* in the absence of TNF*α*. It is not surprising to observe these results given a similar scenario to that observed in *Winnie* when IL-17 is blocked with anti-IL-17 antibodies *in vivo*.^28^

TNF*α* KO mice were previously described as a model mounting a vigorous, disorganized inflammatory response leading to death in response to the injection with heat-killed *Corynebacterium parvum*.^29^ TNF*α* anti-inflammatory properties were previously known, but mainly attributed to the ability to induce T-cell apoptosis.^30^ Here we demonstrate that TNF*α* KO DCs produce large amounts of IL-1*β*, an inflammatory cytokine detectable in patients with active UC.^31^ Our data suggest the existence of a negative feedback between TNF*α* and IL-1*β* as recently noticed by West *et al.*,^32^ indicating IL-1*β* as one of the predictive markers for anti-TNF*α* non-responders during colonic onset of inflammation.

In conclusion, we believe we have described a valuable model that closely represents a cohort of UC patients that are non-responsive to current anti-TNF*α* therapies and also identified a potential biomarker for UC non-responders to anti-TNF*α* therapies. Further research to functionally characterize the role of IL-1*β* would be the next step in targeted combinatorial therapy in UC non-responders patients.

## Material and methods

### Mice

Investigation has been conducted according to national and international guidelines and has been approved by the authors' institutional review board (Organism For Animal Wellbeing – OPBA). The new murine transgenic line TNF*α* KO *Winnie* was created by breeding heterozygous mice from TNF*α* knockout line and heterozygous *Winnie* mice, a murine line established from Dr. Eri’s group at the University of Tasmania. The TNF*α* knockout murine line was purchased from Jackson Laboratories (B6.129S-*Tnftm1Gkl*/J, stock No: 005540; weight: approximately 20 g) (Bar Harbor, ME, USA).

All animal experiments were carried out in accordance with Directive 86/609 EEC enforced by Italian D.L. n. 116 1992, and approved by the Committee on the Ethics of Animal Experiments of Ministero della Salute – Direzione Generale Sanità Animale (768/2015-PR 27/07/2015) and the official RBM veterinarian. Animals were killed if found in severe clinical condition in order to avoid undue suffering.

### Histological examination

Tissue section from the distal colon were fixed in 10% buffered formalin and embedded in paraffin. Sections of 3 *μ*m were stained with H&E. Images were acquired using Leica LMD 6500 (Leica Microsystems, Wetzlar, Germany). The histological score was calculated by adding the values relative to the following parameters: ulceration (0–3), re-epithelization (0–3), transmural inflammation (0–3) and chronic inflammation (0–3).

### RNA extraction and qPCR analysis

Total RNA was isolated from the medial part of the large intestine. The RNA was extracted using TRIzol (Thermo Fisher Scientific, MA, USA) according to the manufacturer’s instructions. Five hundred nanograms of total RNA was reverse transcribed with the High Capacity cDNA Reverse Transcription kit (Thermo Fisher Scientific) by using random primers for cDNA synthesis. Gene expression of *Il1β, Ifnγ, Il12, Il10, Il4* and *Gapdh* was performed with TaqMan gene expression assays (Thermo Fisher Scientific) murine probes: Mm00434228_m1, Mm01168134_m1, Mm01288989_m1, Mm00439614_m1, Mm00445259_m1, Mm99999915_g1, respectively. Real-time analysis was performed on CFX96 System (Biorad, Hercules, CA, USA) and the expression of all target genes was calculated relative to *Gapdh* expression using the ΔΔCt method.

### Multiplex cytokine ELISA

Serum from 5-week-old male mice of each experimental group was analyzed using the Bead-based Multiplex for the Luminex platform (LaboSpace srl, Milano, Italy).

### Generation and culture of murine DCs

BMDCs were obtained from TNF*α* KO mice (5540 line). Briefly, single-cell suspension of BMDCs cells from the tibiae and femurs of 6- to 8-week-old male mice were flushed with 0.5 mM EDTA (Thermo Fisher Scientific), and depleted of red blood cells with ACK lysing buffer (Thermo Fisher Scientific). Cells were plated in a 10 ml dish (1 × 10^6^ cells/ml) in RPMI 1640 (Thermo Fisher Scientific) supplemented with 10% heat-inactivated fetal bovine serum (Thermo Fisher Scientific), 100 U/ml penicillin (Thermo Fisher Scientific), 100 mg/ml streptomycin (Thermo Fisher Scientific), 25 *μ*g/ml rmGM-CSF (Miltenyi Biotec, Bergisch Gladbach, Germany), and 25 *μ*g/ml rmIL-4 (Miltenyi Biotec) at 37 °C in a humidified 5% CO_2_ atmosphere. On day 5 cells were harvested, re-stimulated with new growth factors and plated 1 × 10^6^ cells/ml on 24-well culture plate. On day 7 BMDCs were stimulated with 1 *μ*g/ml of LPS (L6143, Sigma-Aldrich, St Louis, MO, USA) and, at the same time, with TNF*α* standard (40 ng/ml) and anti-TNF*α* or anti-IL-1*β* antibody (400 ng/ml and 80 ng/ml, respectively). Supernatants were collected 24 h after LPS stimulation.

### Western blot analysis

Ileum *were* homogenated as already reported by Terlizzi *et al.*^33^ Homogenized samples were used to examine the expression of NLRP3 (AbCam, UK) and caspase-1 active form (25 kDa; AbCam, Cambridge, UK). Quantitative data were evaluated by means of ImageJ (NIH, Bethesda, MD, USA) software.

### ELISA

Cell culture supernatants were analyzed for IL-1*β* release in triplicate, using an ELISA kit (R&D Systems, Minneapolis, MN, USA) following the manufacturer’ instructions.

### Statistical analysis

Statistical analysis was performed using the Graphpad Prism statistical software release 5.0 for Windows XP. All data were expressed as means±S.E.M. of data obtained from at least three independent experiments. We evaluated statistical significance with two-tailed Student’s *t*-test, one-way ANOVA followed by Tukey’s multiple comparison as post-test and the two-way ANOVA test using the Bonferroni as a post-test for the grouped analysis. Results were considered statistically significant at *P*<0.05.
